# Specific IgG levels before and one month after administration of a booster dose of the diphtheria, tetanus, and pertussis vaccine in 14-year-old adolescents

**DOI:** 10.3389/fimmu.2026.1854969

**Published:** 2026-06-18

**Authors:** Mikhail Kostinov, Ekaterina Prutskova, Irina Mekhantseva, Alexander Cherdantsev, Irina Solovеva, Valentina Polishchuk, Alexander Zhestkov, Anna Khamidulina, Yuliya Dementeva, Anton Kostinov, Isabella Khrapunova, Marina Loktionova, Alla Tarasova, Maria Kvasova, Arseniy Poddubikov, Nidhi Harish Pal, Aristitsa Kostinova

**Affiliations:** 1 I. M. Sechenov First Moscow State Medical University, Moscow, Russia; 2 I. I. Mechnikov Research Institute of Vaccines and Sera, Moscow, Russia; 3 Federal State Budget-Financed Educational Institution of Higher Education, Ulyanovsk State University, Ulyanovsk, Russia; 4 Karaganda Medical University, Karaganda, Kazakhstan; 5 Medical University «Reaviz», Samara, Russia; 6 Federal State Budgetary Educational Institution of Higher Education «Privolzhsky Research Medical University» of the Ministry of Health of the Russian Federation, Nizhniy Novgorod, Russia

**Keywords:** adolescents, immunity to diphtheria, immunity to pertussis, immunity to tetanus, revaccination

## Abstract

**Background:**

The maintenance of durable humoral immunity against diphtheria, tetanus, and Bordetella pertussis during adolescence is critical for long-term protection and epidemiological stability. However, the immunological impact of booster vaccination at 14 years, particularly for pertussis, remains incompletely understood.

**Methods:**

In this prospective, open-label study, 121 healthy 14-year-old adolescents received booster vaccination with Td-M (n = 34), Tdap (n = 51), or Tdap-M (n = 36). Serum IgG antibodies against diphtheria and tetanus toxoids and B. pertussis were quantified by ELISA before and one month after vaccination. Humoral responses were assessed using geometric mean titers and established seroprotection thresholds.

**Results:**

At baseline, all participants exhibited protective immunity to tetanus, while a minority lacked protective antibody levels against diphtheria (1.7%) and pertussis (6.9%). Booster vaccination elicited a robust increase in anti-diphtheria IgG across all groups, with no significant differences between vaccine formulations. All adolescents achieved high levels of tetanus-specific antibodies, indicating strong anamnestic responses, with the highest titers observed in the Td-M group. In contrast, pertussis-specific IgG responses were heterogeneous and showed no overall increase following revaccination, suggesting limited booster-induced humoral expansion in previously primed individuals. Notably, Tdap-M maintained more stable antibody levels compared to Tdap. All vaccines demonstrated good tolerability with only mild adverse events.

**Conclusion:**

Adolescent booster vaccination effectively reinforces humoral immunity against diphtheria and tetanus, reflecting preserved immunological memory. However, the absence of a significant pertussis-specific IgG boost suggests that pre-existing immunity, potentially shaped by prior vaccination and natural exposure, may limit the measurable serological response. These findings question the immunological necessity of routine pertussis revaccination at 14 years and highlight the need for optimization of booster strategies.

## 1 Introduction

The incidence of diphtheria and the carriage of toxigenic Corynebacterium in the Russian Federation (RF) have remained consistently low for more than a decade. Over the past 10 years, the incidence of diphtheria has ranged from 0.001 to 0.003 per 100,000 population. The prevalence of toxigenic Corynebacterium carriage has ranged from 0.001 to 0.008 per 100,000 population (2012). In 2024, no cases of diphtheria or carriage of toxigenic strains were registered, similar to the preceding year (2023) (1).

The persistently low incidence of diphtheria in Russia, with only sporadic cases reported for many years, is largely attributable to the high vaccination coverage in target age groups. Timely administration of primary vaccination and the first booster dose against diphtheria plays a critical role in maintaining population immunity. In 2024, 96.8% of children received the primary vaccination series consisting of three doses by the age of 12 months, exceeding the recommended coverage threshold of 95%. The coverage of timely revaccination against diphtheria among children aged 24 months reached 96.4% nationwide. Maintaining vaccination coverage at or above 95% in each indicator age group remains the key strategy for diphtheria prevention.

Globally, the number of tetanus cases decreased 5.3-fold between 1980 and 2023. In Russia, approximately 800 cases of tetanus were reported annually in the mid-20th century, whereas between 2006 and 2023 the number ranged from 8 to 21 cases per year. Worldwide coverage of the completed three-dose tetanus vaccination series among children was 84-86% during 2018–2023, whereas in Russia it exceeded 96%. Vaccination coverage among adults in Russia exceeds 90%, which is supported by serological monitoring data on population immunity (2).

Pertussis continues to be reported across all population groups. For more than a decade, children younger than 14 years have accounted for approximately 80% of all pertussis cases nationwide. Analysis of age-specific pertussis incidence in 2024 confirmed the persistence of this pattern: children younger than 14 years accounted for 80.6% of cases, adolescents aged 15–17 years for 8.2%, and adults for 11.2% (1, 3, 4). Vaccination coverage against pertussis in the target age groups (3–24 months) has exceeded the recommended level of 95% over the past decade. In 2024, 96.6% of children in the Russia were vaccinated against pertussis on schedule by 12 months of age, and 96.3% received timely revaccination at 24 months, which is comparable to the data reported in 2023.

Immunity to diphtheria and tetanus in adolescents largely depends on adherence to the national immunization schedule. In Russia, as in many other countries, mass vaccination with a combined diphtheria–tetanus–pertussis vaccine (DTP) was introduced in 1964-1965. The primary vaccination course includes four doses administered between the ages of 3 and 18–24 months. Since the late 1960s and early 1970s, routine second and third booster vaccinations against diphtheria and tetanus have been administered at 7 years (previously 6–7 years) and 14 years of age using reduced-dose diphtheria and tetanus toxoids (Td-M) (5, 6).

In the Russian Federation, vaccination against pertussis is limited to four doses administered during early childhood using DTP vaccines. Until 2021, whole-cell pertussis vaccines were predominantly used, followed by the gradual replacement with acellular pertussis components. According to the vaccine instructions, the whole-cell pertussis vaccine (DTwP) is approved for use from 3 months to 5 years of age, whereas the acellular pertussis vaccine (DTaP) can be administered from 3 months to 7 years of age.

Despite the routine monitoring of population immunity to vaccine-preventable infections conducted by public health authorities, it remains important to evaluate the current level of specific antibodies against diphtheria, tetanus, and pertussis in adolescents. In the present study, we assessed antibody levels in 14-year-old children before and one month after administration of the third booster dose of a combined vaccine containing different quantities of diphtheria and tetanus toxoids and a second booster dose of the pertussis component. The evaluation of immunity to these infections is particularly important because maintaining robust protection against diphtheria and tetanus contributes to the continued stability of the epidemiological situation. In the case of pertussis, the assessment allows evaluation of the effectiveness of booster vaccination during adolescence, considering that children previously received the fourth dose of whole-cell pertussis vaccine (DTwP) at an early age (18–24 months).

Maintaining population immunity against vaccine-preventable infections is especially important under current conditions of increased global mobility and migration, which may contribute to the emergence of localized outbreaks of infectious diseases (7–13).

Furthermore, adolescents frequently remain in organized social settings, including secondary schools, universities, and military service, where close contact facilitates the rapid transmission of infectious diseases (14–16). It should also be noted that adolescents demonstrate an unfavorable trend in terms of increasing overall infectious morbidity (17–19).

Therefore, the aim of this study was to evaluate the levels of specific IgG antibodies before and after administration of a booster dose of diphtheria–tetanus–pertussis vaccine in adolescents aged 14 years.

## 2 Materials and methods

### 2.1 Study design

The study was designed as a single-center, prospective, open-label, comparative study with parallel groups of 14-year-old adolescents. The primary objective of the study was to assess the intensity of specific IgG responses to diphtheria, tetanus, and pertussis one month after administration of different vaccine formulations.

**Figure 1:**
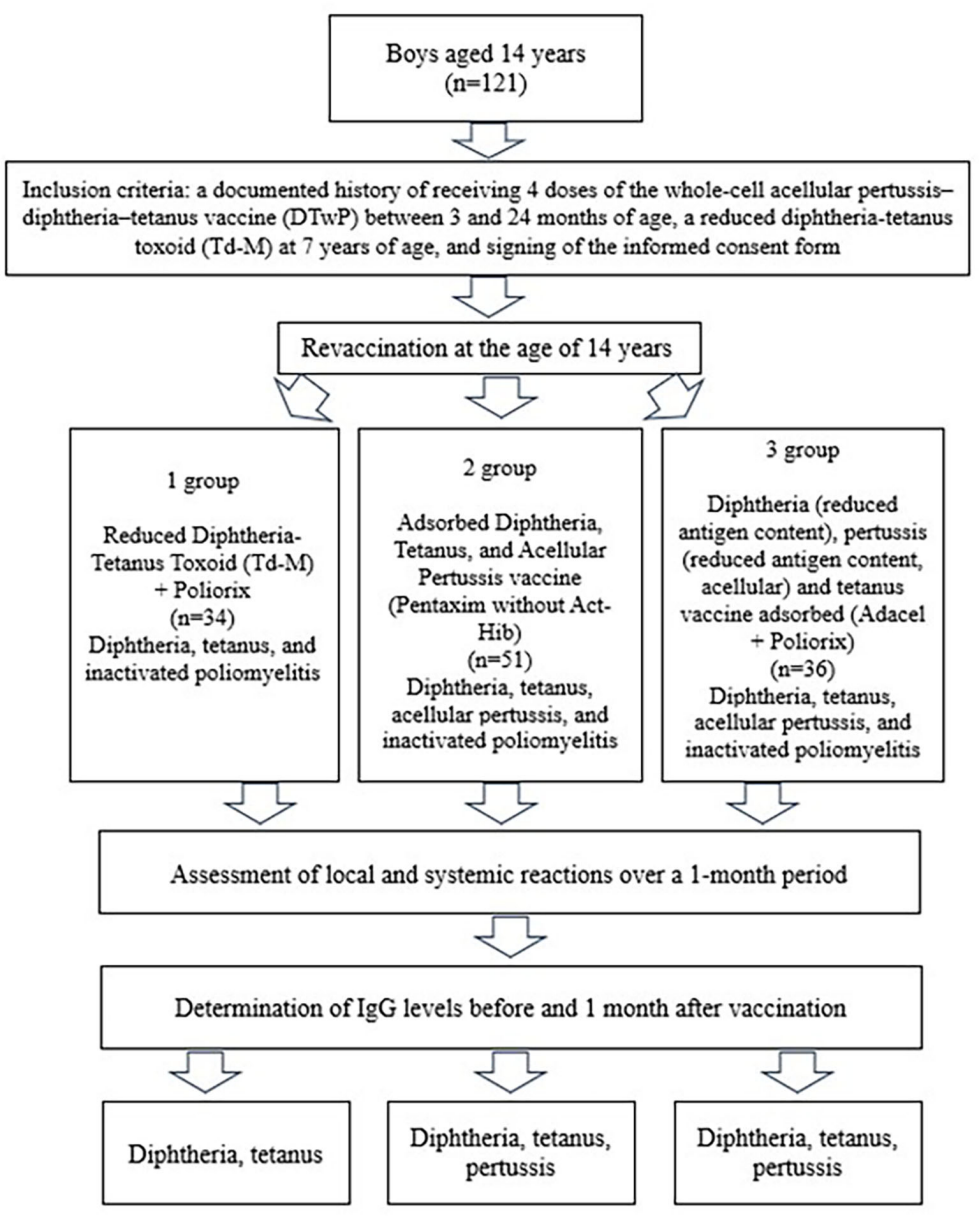
Study design.

The study included 121 adolescents (boys) aged 14 years. According to their vaccination history, all participants had previously received four doses of pertussis-containing vaccine (the last dose administered at 18–24 months of age) and six doses of diphtheria–tetanus vaccine (the last dose administered at 6–7 years of age) (**Figure 1**).

The primary objective of the study was to assess the status of post-vaccination antitoxic immunity to diphtheria and tetanus in adolescents aged 14 years prior to administration of the third booster dose against these infections, as well as to evaluate the level of protection against pertussis 12 years after the first booster dose of the whole-cell pertussis vaccine administered as part of the DTwP vaccination series.

The secondary objective was to demonstrate the development of specific antibodies by Day 30 in three groups of adolescents vaccinated with different vaccine formulations: adsorbed diphtheria and tetanus toxoid with reduced antigen content (Td-M), adsorbed diphtheria, tetanus, and acellular pertussis vaccine (Tdap) and diphtheria (reduced antigen content), pertussis (reduced antigen content, acellular) and tetanus vaccine adsorbed (Tdap-M), which differ in antigen composition and quantity per vaccine dose. In addition, the study aimed to characterize the clinical tolerability of vaccination by assessing the occurrence of local and systemic reactions during Days 1–8 following immunization.

### 2.2 Ethical considerations

The study was conducted between 2016 and 2020 at the following institutions: Ulyanovsk State University, the I.I. Mechnikov Research Institute of Vaccines and Sera, and Sechenov First Moscow State Medical University (Sechenov University), Ministry of Health of the Russian Federation.

The study protocol was approved by the Local Ethics Committee of Ulyanovsk State University (Protocol №11, November 23, 2016). The study was performed in accordance with the approved study protocol and complied with the national standard of the Russian Federation GOST R 52379-2005 “Good Clinical Practice” and with the international principles of Good Clinical Practice (GCP). The participants’ legal guardians/next of kin provided written informed consent to participate in this study. Written informed consent was obtained from the minors’ legal guardian/next of kin for the publication of any potentially identifiable data included in this article.

All potential participants underwent screening according to the predefined inclusion and exclusion criteria. *Inclusion criteria* were: age of 14 years; ability to comply with the study protocol; attendance at scheduled study visits and blood sampling for laboratory testing; and provision of written informed consent to participate in the clinical study. *Exclusion criteria* included: leukemia, malignant neoplasms, or a history of positive test results for HIV infection or hepatitis B or C; administration of immunoglobulin preparations or blood transfusion within three months prior to study initiation; significant delay in physical development; use of immunosuppressive or other immunomodulatory medications within six months prior to the study (for more than 14 days); confirmed or suspected immunosuppressive or immunodeficiency conditions; severe congenital abnormalities or serious chronic diseases, including disorders of the respiratory, renal, cardiovascular, or nervous systems, psychiatric diseases, or metabolic disorders confirmed by medical history or clinical examination; acute infectious and/or non-infectious diseases at the time of enrollment; and a history of severe post-vaccination adverse reactions. These criteria were established to ensure the safety of the participants and the reliability of the study results.

### 2.3 Study participants

All enrolled adolescents were recruited from an educational institution and belonged to Health Group I, a classification used for children considered to be completely healthy. This category includes individuals without chronic diseases, significant functional disorders, or known risk factors for their development, and who do not require regular medical follow-up for any medical conditions. It represents the most favorable health category and implies no restrictions on participation in sports or other physical activities.

A review of the preventive vaccination records (Form 063/u – official preventive vaccination record used in the Russian Federation) confirmed that all adolescents had previously been vaccinated according to the schedule established by the National Immunization Program. Specifically, all participants had received three doses of the Tdap vaccine between the ages of 3 and 12 months, followed by the first booster dose at 18–24 months of age. In addition, all participants had received one dose of reduced Diphtheria-Tetanus Toxoid (Td-M) vaccine at 7 years of age (second booster vaccination against diphtheria and tetanus)

### 2.4 Vaccines

The vaccines used in the study and their combinations are presented in **Table 1**.

**Table 1:** Composition of vaccines used for vaccination of 14-year-old adolescents. .

Vaccines and study groups	Vaccine composition (1 dose = 0.5 mL)									
	DT Lf(ME)	TT Lf(ME)	PT (мcg)	FHA (мcg)	FIM 2.3(мcg)	PRN (мcg)	IPV 1(units)	IPV 2(units)	IPV 3(units)	Al(OH)_3_ (мg)
1 group (n=34) Reduced Diphtheria-Tetanus Toxoid (Td-M) + Poliorix	5 (5)	5 (20)	–	–	–	–	–	–	–	0.55
	–	–	–	–	–	–	40	8	32	–
2 group (n=51) Pentaxim without Act-Hib	30 (≥30)	10 (≥40)	25	25	–	–	29	7	26	0.3
3 group (n=36) Adacel + Poliorix	2 (2)	5 (20)	2.5	5	5	3	–	–	–	0.33
	–	–	–	–	–	–	40	8	32	–

DT, diphtheria toxoid; TT, tetanus toxoid; PT, pertussis toxoid; FHA, filamentous hemagglutinin; FIM, fimbrial agglutinogens types 2 and 3; PRN, pertactin; IPV1–3, inactivated poliovirus types 1–3; Al(OH)_3_, aluminum hydroxide; Act-Hib, Haemophilus influenzae type b vaccine; (+), simultaneous administration of two vaccines.

As shown in **Table 1**, adolescents in the first group (n = 34) received intramuscular vaccination for the prevention of diphtheria, tetanus, and poliomyelitis. The Reduced Diphtheria-Tetanus vaccine (Td-M), 0.5 mL (Anatoxinum diphtherico-tetanicum purificatum adsorptum cum quantitate minore antigenorum fluidum; manufacturer: JSC “NPO Microgen”, Russia) (20), was administered together with the inactivated poliomyelitis vaccine Poliorix^®^ (poliomyelitis vaccine (inactivated)), 0.5 mL (manufacturer: GlaxoSmithKline Biologicals, Belgium). The vaccines were administered intramuscularly into the upper third of different arms.

The second group (n=51) received intramuscularly a vaccine for the prevention of diphtheria and tetanus (adsorbed), pertussis (acellular), and poliomyelitis (inactivated) (diphtheria and tetanus adsorbed, pertussis acellular, poliomyelitis inactivated) - Pentaxim (Pentaxim) without the Haemophilus influenzae type b vaccine conjugated component (Tdap) (manufacturer: Sanofi Winthrop Industrie, France).

The third group (n=36) of adolescents received intramuscularly a vaccine for the prevention of diphtheria (reduced antigen content), pertussis (reduced antigen content, acellular), and tetanus, adsorbed (diphtheria (reduced antigen content), pertussis (reduced antigen content, acellular) and tetanus vaccine adsorbed) - Adacel^®^ (Adacel^®^) (Tdap-M) (manufacturer: Sanofi Pasteur Limited, Canada), and for the prevention of poliomyelitis - an inactivated poliomyelitis vaccine (poliomyelitis vaccine (inactivated)) - Poliorix^®^, 0.5 mL (manufacturer: GlaxoSmithKline Biologicals, s.a., Belgium), administered into different upper thirds of the shoulder.

Vaccination was performed in accordance with ethical standards and with the recommendations of the World Health Organization (WHO) and the Ministry of Health of the Russian Federation.

### 2.5 Assessment of post-vaccination reactions and safety

Adolescents were observed for 40 minutes after vaccination to monitor the occurrence of any immediate adverse reactions. All changes in health status were recorded in an observation diary, which was completed daily throughout the first month of follow-up. The list of monitored symptoms included an assessment of general condition (satisfactory or unsatisfactory), local reactions at the injection site (pain, hyperemia, and induration), and systemic symptoms such as fever, cough, pharyngitis, increased fatigue, arthralgia, myalgia, headache, dizziness, nausea, vomiting, abdominal pain, and diarrhea. The severity of post-vaccination reactions was assessed using a scoring scale (**Table 2**).

**Table 2:** Assessment of the severity of post-vaccination reactions in adolescents.

*Local reactions at the injection site*	
0 – Absent	No symptoms
1 – Mild	Hyperemia with a diameter ≤50 mm or induration with a diameter ≤25 mm
2 – Moderate	Hyperemia with a diameter >50 mm or induration with a diameter of 26–50 mm
3 – Severe	Induration with a diameter >50 mm
Systemic reactions	
0 – Absent	No symptoms
1 – Mild	Presence of mild symptoms
2 – Moderate	Symptoms noticeably interfering with normal daily activities
3 – Severe	Symptoms preventing normal daily activities
Fever	
0 – Absent	≤ 37°С
1 – Mild	>37.0 °C to ≤37.5 °C
2 – Moderate	> 37.6°С to £ 38.5°С
3 – Severe	> 38.6°С

### 2.6 Biological sample collection

Venous blood samples were collected before vaccination and one month after vaccine administration. To ensure standardization of laboratory procedures and obtain reliable study results, blood sampling was performed in the morning, prior to physical activity, either in the fasting state or after a light breakfast. Venous blood was collected into vacuum tubes (Improvacuter) containing a clot activator and transported to the laboratory. After centrifugation at 3000 rpm, serum was separated and stored in Eppendorf tubes at −80 °C in a cryogenic freezer for subsequent serological analysis.

### 2.7 Assessment of vaccine-induced antibodies

Serum IgG antibodies against diphtheria toxoid were measured using the Anti-Diphtheria Toxoid ELISA IgG kit (Euroimmun, Germany). According to the manufacturer’s instructions, antibody levels were interpreted as follows: <0.01 IU/mL (unprotected), 0.01–0.09 IU/mL (equivocal), 0.1–1.0 IU/mL (protected – sufficient), and >1.0 IU/mL (high, long-term protection).

Serum IgG antibodies against tetanus toxoid were determined using the Anti-Tetanus Toxoid ELISA IgG kit (Euroimmun, Germany), which measures antitoxic antibody levels for the evaluation of immunity to tetanus. The interpretation of antibody levels was as follows: <0.01 IU/mL (unprotected), 0.01–0.09 IU/mL (equivocal), 0.1–1.0 IU/mL (protected – sufficient), and >1.0 IU/mL (high, long-term protection).

IgG antibodies against Bordetella pertussis were measured in plasma using the Bordetella pertussis IgG ELISA kit (DRG Instruments GmbH, Germany). According to the manufacturer’s instructions, the results were interpreted as follows: Negative <9 DU; Gray zone 9–11 DU; Positive >11 DU.

All laboratory analyses were performed using certified equipment of the Core Facility Center of the I.I. Mechnikov Research Institute of Vaccines and Sera.

### 2.8 Statistical analysis

The statistical significance of changes in the distribution of IgG levels against diphtheria toxoid, tetanus toxoid, and Bordetella pertussis in adolescents over time was evaluated using McNemar’s test. Between-group differences were assessed using the χ² test with Monte Carlo simulation (10.000 permutations). Statistical analyses were performed using the stats 4.5.1 package (functions mcnemar.test and chisq.test). Data processing and analysis were conducted in RStudio, using R version 4.5.1 (2025-06–13 ucrt).

In addition to the χ² test, standardized residual analysis was used to evaluate the contribution of individual cells in contingency tables. Standardized residuals were calculated as the difference between observed and expected values normalized by their variance. For interpretation, residual values exceeding ±2 were considered indicative of a significant deviation between observed and expected frequencies. Positive residuals indicated an excess, whereas negative residuals indicated a deficit of responses relative to the independence model.

For the statistical analysis of geometric mean titers (GMTs), antibody titers were log-transformed using a base-2 logarithm (log_2_). Between-group comparisons of vaccine immunogenicity (Td-M, Tdap and Tdap-M) at each time point were performed using the Student’s t-test for independent samples. Within-group changes in antibody titers over time were evaluated using the paired Student’s t-test. Differences were considered statistically significant at p < 0.05.

## 3 Result

### 3.1 IgG antibodies against diphtheria toxoid

**Figure 2:**
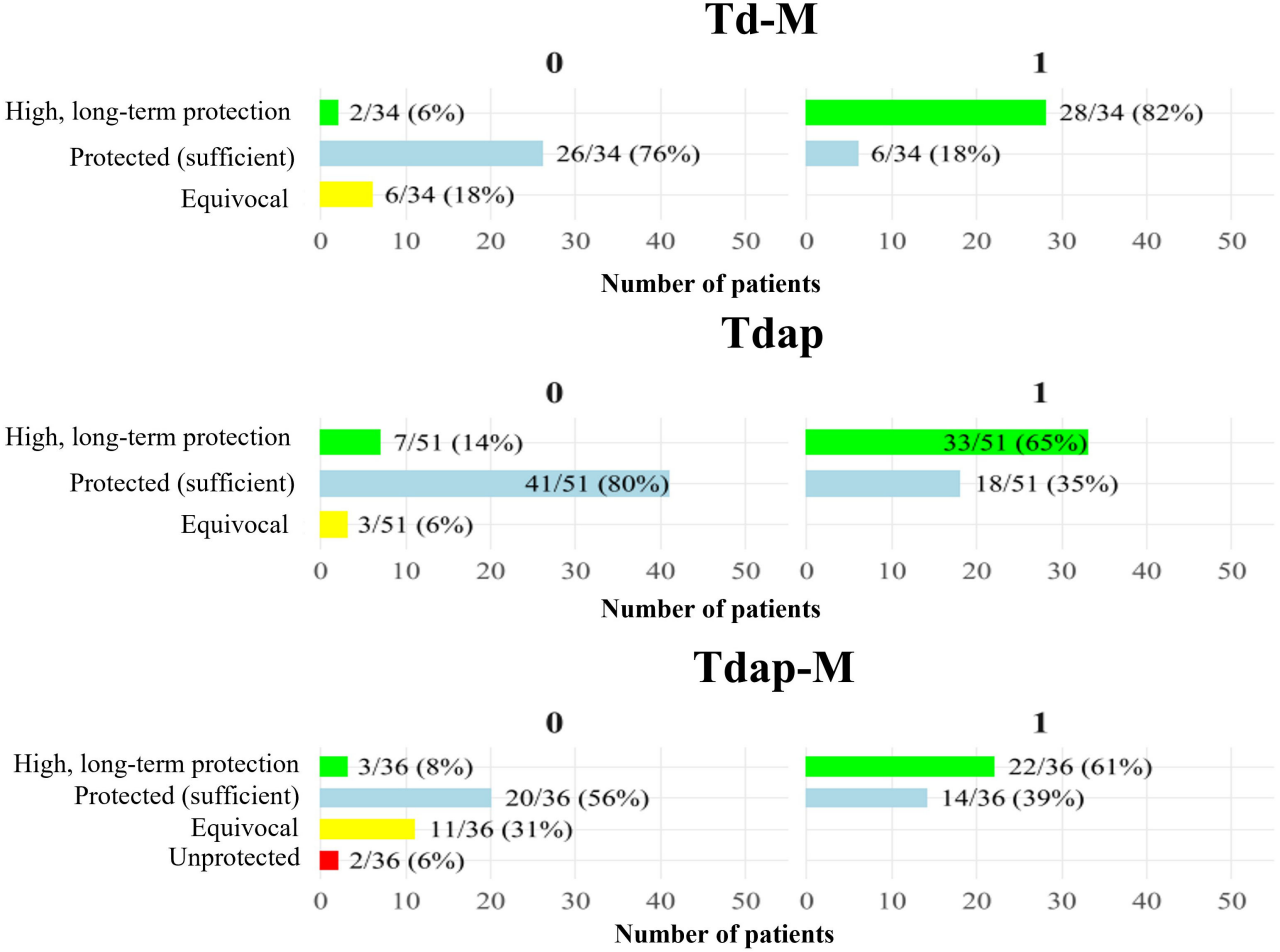
Distribution of IgG levels against diphtheria toxoid in adolescents before and one month after revaccination with different vaccines. Data are presented as n/N (%).

The distribution of IgG antibody levels against diphtheria toxoid among revaccinated adolescent groups was assessed at baseline before vaccine administration (Month 0) and one month after revaccination (Month 1) using categorical serological outcomes (**Table 3**, **Figure 2**).

**Table 3:** Distribution of IgG levels against diphtheria toxoid in adolescents before and one month after revaccination with different vaccines.

Protection category	Month 0 (baseline)	Month 1 (post-vaccination)	p*
Td-М (n=34)			
High, long-term protection, >1.0 IU/mL	2/34 (5.9%)	28/34 (82.4%)	< 0.001
Protected (sufficient), 0.1–1.0 IU/mL	26/34 (76.5%)	6/34 (17.6%)	< 0.001
Equivocal, 0.01–0.09 IU/mL	6/34 (17.6%)	–	0.041
Tdap (n=51)			
High, long-term protection, >1.0 IU/mL	7/51 (13.7%)	33/51 (64.7%)	< 0.001
Protected (sufficient), 0.1–1.0 IU/mL	41/51 (80.4%)	18/51 (35.3%)	<0.001
Equivocal, 0.01–0.09 IU/mL	3/51 (5.9%)	–	0.25
Tdap-М (n=36)			
High, long-term protection, >1.0 IU/mL	3/36 (8.3%)	22/36 (61.1%)	< 0.001
Protected (sufficient), 0.1–1.0 IU/mL	20/36 (55.6%)	14/36 (38.9%)	0.26
Equivocal, 0.01–0.09 IU/mL	2/36 (5.6%)	–	0.48
High, long-term protection, >1.0 IU/mL	11/36 (30.6%)	–	0.003
P**	0.011	0.112	

Data are presented as n/N (%).

* Exact McNemar’s test for paired samples.

** P-values were calculated using the χ² test with Monte Carlo simulation (B = 10.000).

Values in bold indicate cells with standardized residuals >2 or <−2.

At baseline, the groups demonstrated statistically significant differences in the structure of immunity to diphtheria (χ², p = 0.011). Only 2 of 36 adolescents (5.6%) scheduled for revaccination with Tdap-M had antibody levels below the protective threshold (<0.01 IU/mL). One month after revaccination, IgG levels against diphtheria toxoid did not differ significantly between the groups (χ², p = 0.112). However, analysis of Pearson’s standardized residuals revealed that adolescents receiving Td-М had a significantly higher frequency of developing anti-diphtheria IgG levels corresponding to “high, long-term protection” (std. res. = 2.03), accompanied by a reduced proportion of individuals with “sufficient protection” (std. res. = −2.03) compared with adolescents revaccinated with Tdap and Tdap-М. Thus, administration of Td-М demonstrated a tendency toward the development of a stronger early immune response to diphtheria compared with the other revaccination groups.

**Figure 3:**
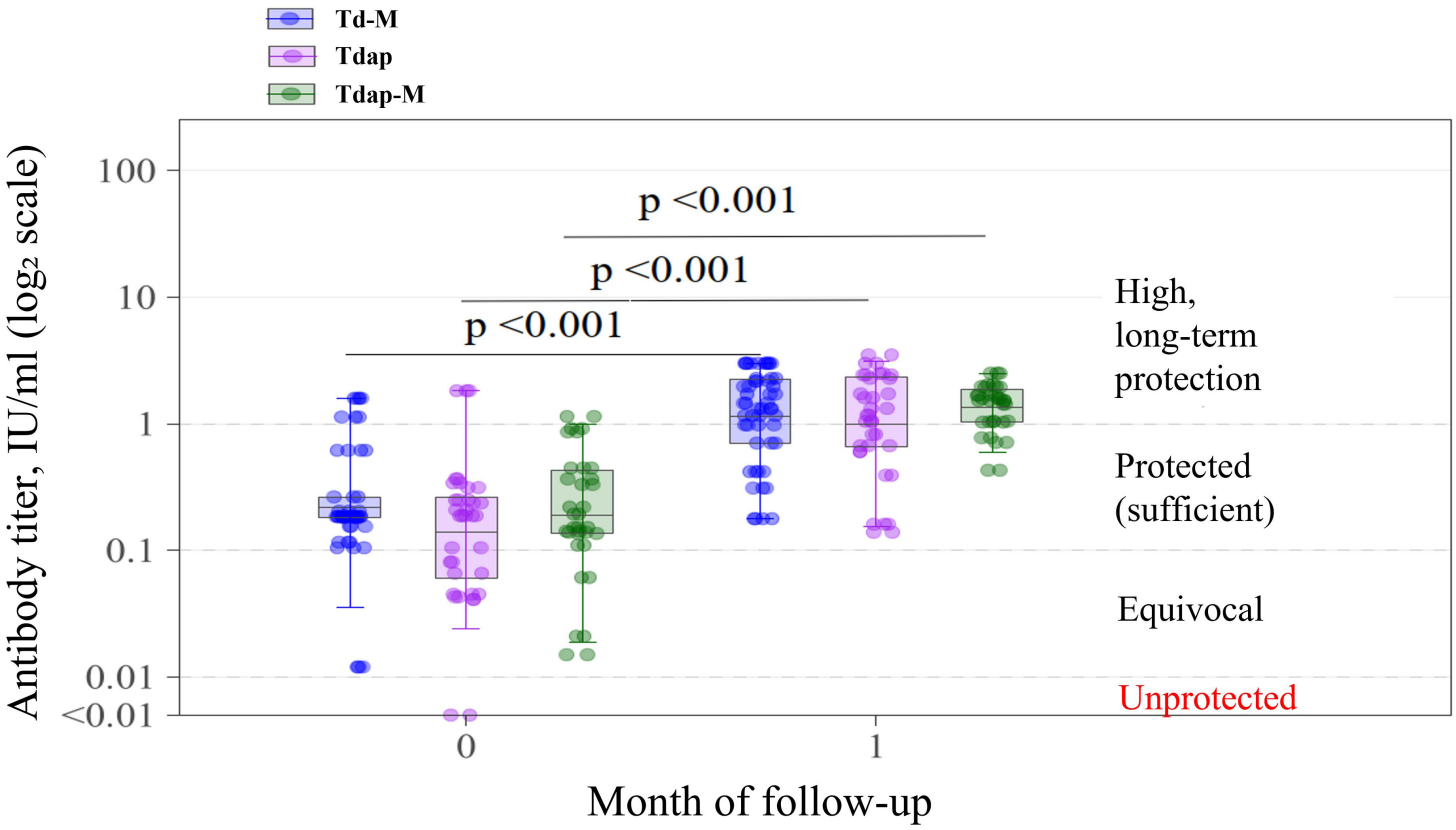
Geometric mean titers (GMT) of IgG antibodies against diphtheria toxoid in adolescents before and one month after revaccination with different vaccines. Data are presented as boxplots: the center line represents the geometric mean titer (GMT), the box boundaries indicate the 25th–75th percentiles (Q1–Q3), and the whiskers represent the 5th–95th percentiles. Points on the plots represent individual values. Only statistically significant differences (p < 0.05) are indicated. Assessment of immunity to diphtheria: <0.01 IU/mL (unprotected), 0.01–0.09 IU/mL (equivocal), 0.1–1.0 IU/mL (protected – sufficient), >1.0 IU/mL (high, long-term protection).

Analysis of geometric mean titers (GMTs) of IgG antibodies against diphtheria toxoid showed that baseline antibody levels were within the protective range in all study groups and ranged from 0.1 to 0.2 IU/mL (**Table 4**, **Figure 3**). One month after vaccination (Td-М, Tdap, Tdap-М), a statistically significant increase in antibody levels was observed (p < 0.001, paired t-test). The magnitude of the response was comparable across all groups, with GMT increasing to approximately 1.0–1.3 IU/mL. No significant differences between vaccines were detected in between-group comparisons (p > 0.05, Holm correction).

**Table 4:** Geometric mean titers (GMT) of IgG antibodies against diphtheria toxoid in adolescents before and one month after revaccination with different vaccines.

Vaccine	n	Month 0 (baseline)	Month 1 (post-vaccination)	p*
		GMT IU/mL (log2 ± SD)	GMT IU/mL (log2 ± SD)	
Td-М	34	0.2 (log2 -2.41 ± 1.71)	1.3 (log2 0.43 ± 0.66)	<0.001
Tdap	51	0.2 (log2 -2.20 ± 1.55)	1.2 (log2 0.21 ± 1.25)	<0.001
Tdap-М	36	0.1 (log2 -2.96 ± 2.27)	1.0 (log2 0.00 ± 1.40)	<0.001
All vaccines	121	0.2 (log2 -2.49 ± 1.85)	1.2 (log2 0.21 ± 1.17)	<0.001
p**		ns	Ns	

Data are presented as GMT IU/mL (log_2_(GMT) ± SD [log_2_(titer)]).

* Paired t-test.

** Between-group comparison: two-sample t-test. Holm correction for multiple comparisons was applied separately for each month. n.s. – not significant.

Combined analysis of all vaccinated adolescents (n = 121) also confirmed a significant increase in IgG levels against diphtheria toxoid (from 0.2 to 1.2 IU/mL, p < 0.001). Thus, all three vaccine formulations induced a comparable specific immune response to diphtheria toxoid in adolescents.

### 3.2 IgG antibodies against tetanus toxoid

**Figure 4:**
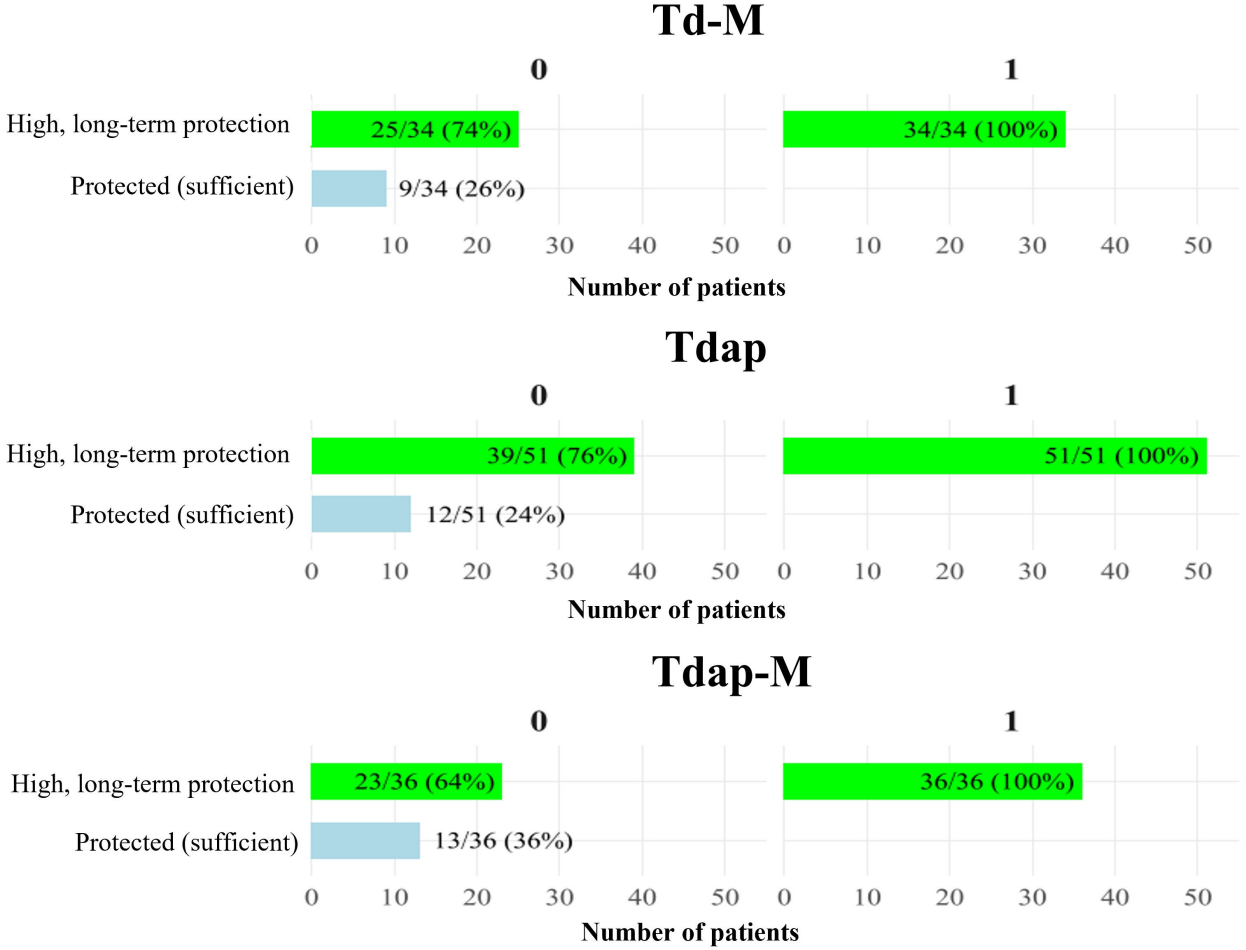
Distribution of IgG antibodies against tetanus toxoid in adolescents before and one month after revaccination with different vaccines. Data are presented as n/N (%).

At baseline (Month 0), 100% of adolescents had protective levels of IgG antibodies against tetanus toxoid. The proportion of individuals with high antibody levels (>1.0 IU/mL), corresponding to high, long-term protection, predominated in all three groups, ranging from 63.9% to 76.5% (**Table 5**, **Figure 4**).

**Table 5:** Distribution of IgG antibodies against tetanus toxoid in adolescents before and one month after revaccination with different vaccines.

Protection category	Month 0 (baseline)	Month 1 (post-vaccination)	p*
Td-М (n=34)			
High, long-term protection, >1.0 IU/mL	25/34 (73.5%)	34/34 (100%)	0.008
Protected (sufficient), 0.1–1.0 IU/mL	9/34 (26.5%)	-	0.008
Tdap (n=51)			
High, long-term protection, >1.0 IU/mL	39/51 (76.5%)	51/51 (100%)	0.001
Protected (sufficient), 0.1–1.0 IU/mL	12/51 (23.5%)	-	0.001
Tdap-М (n=36)			
High, long-term protection, >1.0 IU/mL	23/36 (63.9%)	36/36 (100%)	< 0.001
Protected (sufficient), 0.1–1.0 IU/mL	13/36 (36.1%)	-	< 0.001
P**	ns	Ns	

Data are presented as n/N (%).

* Exact McNemar’s test for paired samples.

** P-values were calculated using the χ² test with Monte Carlo simulation (B = 10.000); n.s. – not significant.

Between-group comparison of baseline immunity to tetanus revealed no statistically significant differences among adolescents receiving Td-М, Tdap, and Tdap-М (χ², p = 0.425).

One month after the scheduled revaccination at 14 years of age, performed according to the National Immunization Schedule, all three groups demonstrated a 100% rate of high, long-term protection. Due to the absence of variability in the outcome, further statistical comparison was not performed.

**Figure 5:**
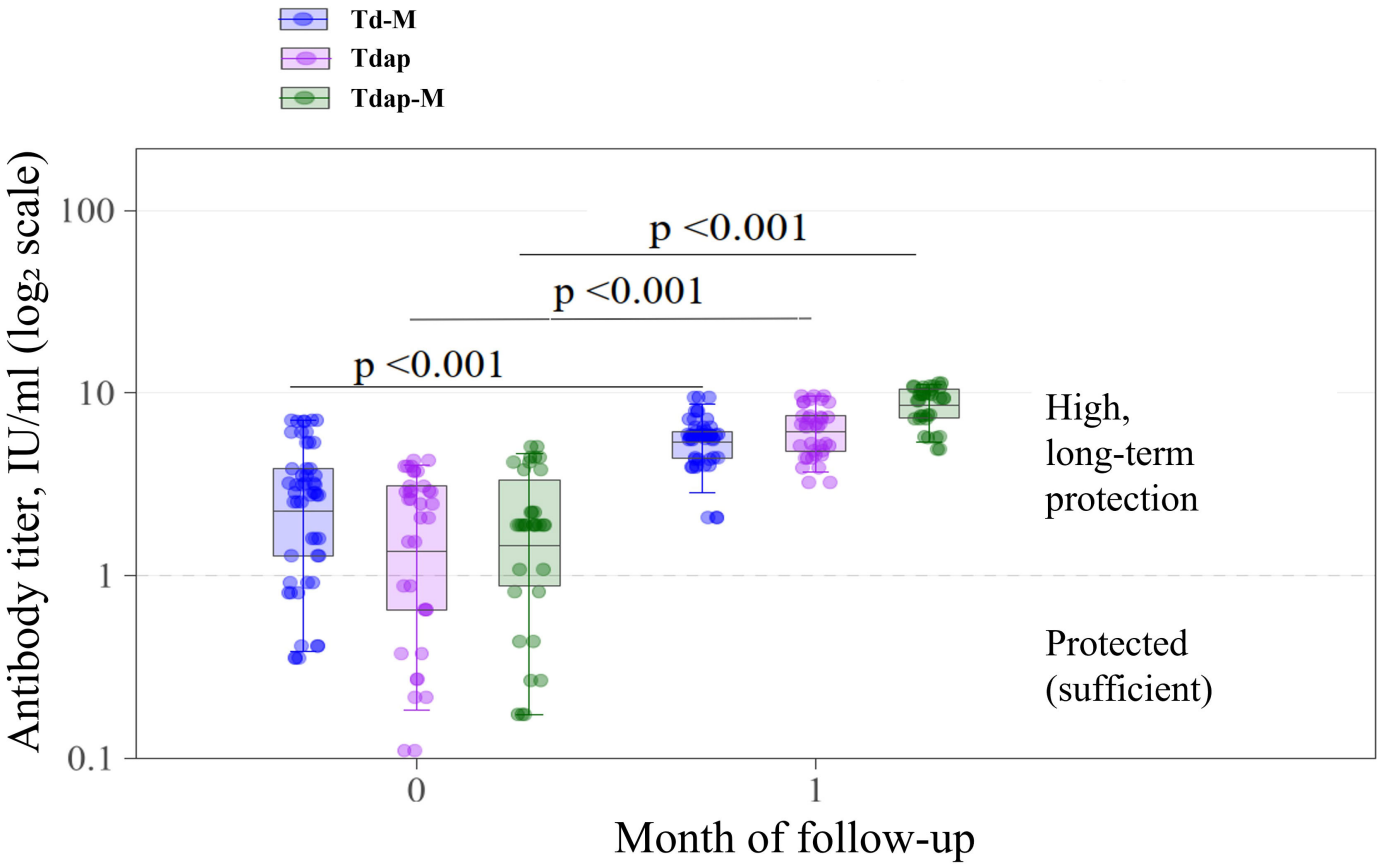
Geometric mean titers (GMT) of IgG antibodies against tetanus toxoid in adolescents before and one month after revaccination with different vaccines. Data are presented as boxplots: the center line represents the geometric mean titer (GMT), the box boundaries indicate the 25th–75th percentiles (Q1–Q3), and the whiskers represent the 5th–95th percentiles. Points represent individual values. Only statistically significant within-vaccine differences (p < 0.05) are indicated.

Analysis of geometric mean titers (GMTs) of IgG antibodies against tetanus toxoid showed that baseline antibody levels were comparable across all adolescent groups at study entry (p > 0.05) (**Table 6**; **Figure 5**). One month after revaccination, a significant increase in antibody titers was observed in all groups, reaching a level corresponding to high seroprotection. The highest GMT was observed in adolescents who received Td-М, reaching 8.5 IU/mL (log_2_: 3.09 ± 0.37). This value was significantly higher than those observed in adolescents revaccinated with Tdap (5.4 IU/mL, log_2_: 2.44 ± 0.48; p < 0.001) and Tdap-М (6.1 IU/mL, log_2_: 2.61 ± 0.46; p < 0.001). A statistically significant increase in GMTs of IgG antibodies against tetanus toxoid from baseline to Month 1 was observed within each study group (p < 0.001; **Table 6**).

**Table 6:** Geometric mean titers (GMT) of IgG antibodies against tetanus toxoid in adolescents before and one month after revaccination with different vaccines.

Vaccine	n	Month 0 (baseline)	Month 1 (post-vaccination)	p*
		GMT IU/mL (log2 ± SD)	GMT IU/mL (log2 ± SD)	
Td-М	34	1.5 (log2 0.54 ± 1.49)	8.5 (log2 3.09 ± 0.37)	<0.001
Tdap	51	2.3 (log2 1.17 ± 1.31)	5.4 (log2 2.44 ± 0.48)	<0.001
Tdap-М	36	1.4 (log2 0.44 ± 1.64)	6.1 (log2 2.61 ± 0.46)	<0.001
All vaccines	121	1.7 (log2 0.78 ± 1.49)	6.4 (log2 2.67 ± 0.52)	<0.001
P**		ns	PTd-М – Tdap<0.001 PTd-М – Tdap-М <0.001	

Data are presented as geometric mean titers (GMT), IU/mL (log_2_(GMT) ± SD [log_2_(titer)]).

* Paired t-test.

** Between-group comparison: two-sample t-test. Holm correction for multiple comparisons was applied separately for each month; n.s., not significant.

### 3.3 IgG antibodies against Bordetella pertussis

**Figure 6:**
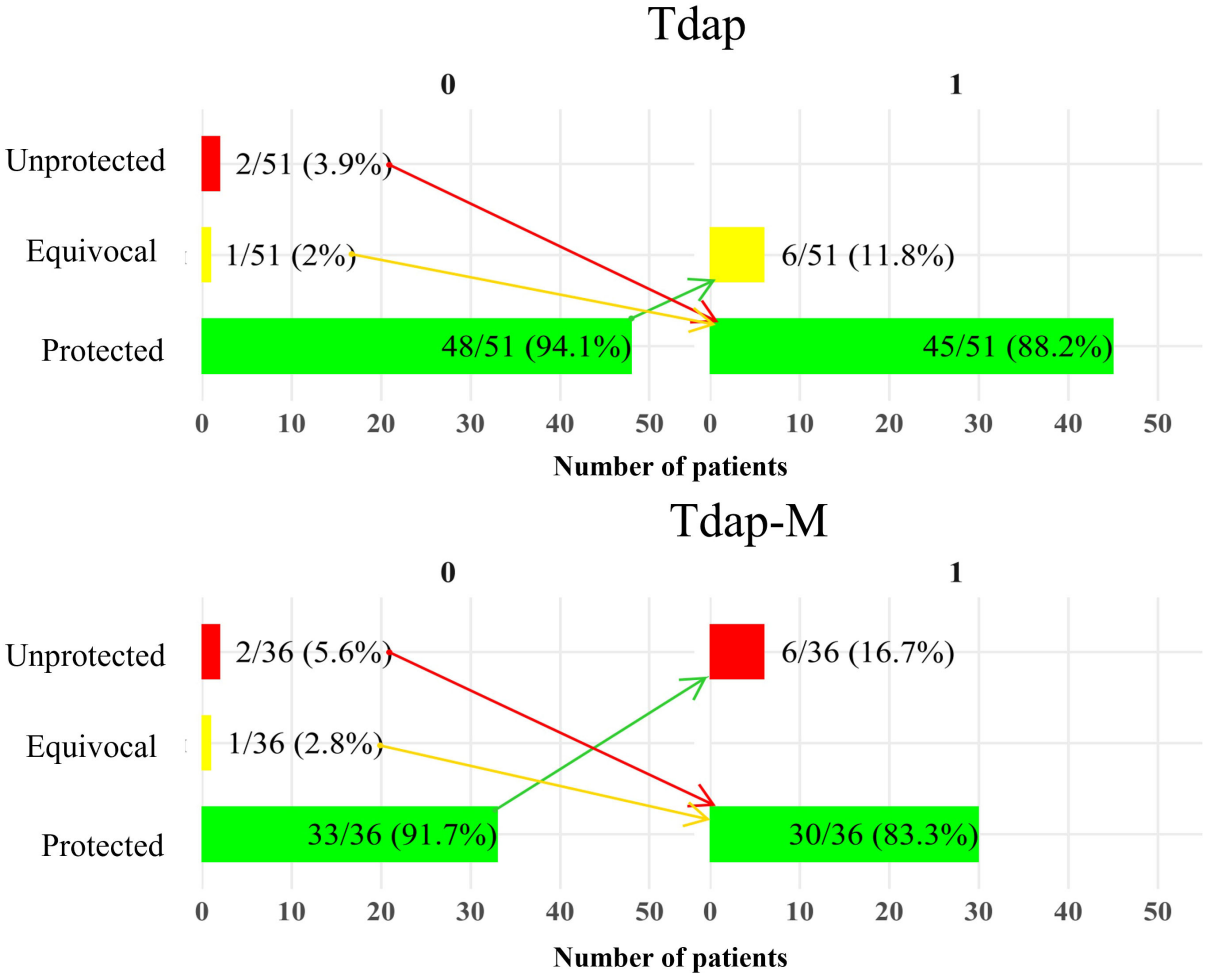
Distribution of IgG antibodies against Bordetella pertussis in adolescents before and one month after revaccination with different vaccines. Data are presented as n/N (%).

At baseline (**Table 7**; **Figure 6**), IgG antibody levels against Bordetella pertussis were comparable between adolescents scheduled for revaccination with Tdap and Tdap-М (p > 0.05). A very high baseline seropositivity rate was observed in both groups, reaching 94.1% and 91.7%, respectively, indicating protective antibody levels (>11 DU). The proportion of unprotected individuals was minimal (3.9% and 5.6%, respectively). Despite revaccination, no statistically significant changes in the distribution of IgG levels against Bordetella pertussis across protection categories were observed within each group (McNemar’s test, p > 0.05 for all categories).

**Table 7:** Distribution of IgG antibodies against Bordetella pertussis in adolescents before and one month after revaccination with different vaccines. .

Protection category	Month 0 (baseline)	Month 1 (post-vaccination)	p*
Tdap (n=51)			
Unprotected, <9 DU	2/51 (3.9%)	–	0.48
Equivocal, 9–11 DU	1/51 (2%)	6/51 (11.8%)	0.13
Protected, >11 DU	48/51 (94.1%)	45/51 (88.2%)	0.50
Tdap-M (n=36)			
Unprotected, <9 DU	2/36 (5.6%)	6/36 (16.7%)	0.29
Equivocal, 9–11 DU	1/36 (2.8%)	–	1.00
Protected, >11 DU	33/36 (91.7%)	30/36 (83.3%)	0.50
p**	ns	< 0.001	

Data are presented as n/N (%).

* Exact McNemar’s test for paired samples.

** P-values were calculated using the χ² test with Monte Carlo simulation (B = 10.000).

Values in bold indicate cells with standardized residuals >2 or <−2.

In the group of adolescents vaccinated with Tdap, no seronegative individuals (<9 DU) were detected one month after vaccination. However, a subgroup of adolescents (11.8%) demonstrated equivocal antibody levels (9–11 DU) that did not reach the protective threshold for IgG against Bordetella pertussis. Prior to revaccination, these individuals belonged to the subgroup with protective antibody levels (>11 DU) (**Figure 6**).

In the group revaccinated with Tdap-М, no adolescents with equivocal results were observed one month after vaccination. However, a subgroup (16.7%) exhibited IgG levels against Bordetella pertussis below the protective threshold, although these individuals had protective antibody levels prior to vaccination (**Figure 6**).

This mechanism has not yet been fully studied, but it can be hypothesized that the presence of a high level of post-infection immunity could have contributed to the suppression of the synthesis of specific antibodies to B.pertussis. Alternatively, it is possible that we have administered in a refractory phase of the immune response associated with the formation of antibodies (the phenomenon of immunological refractoriness (tolerance)).

**Figure 7:**
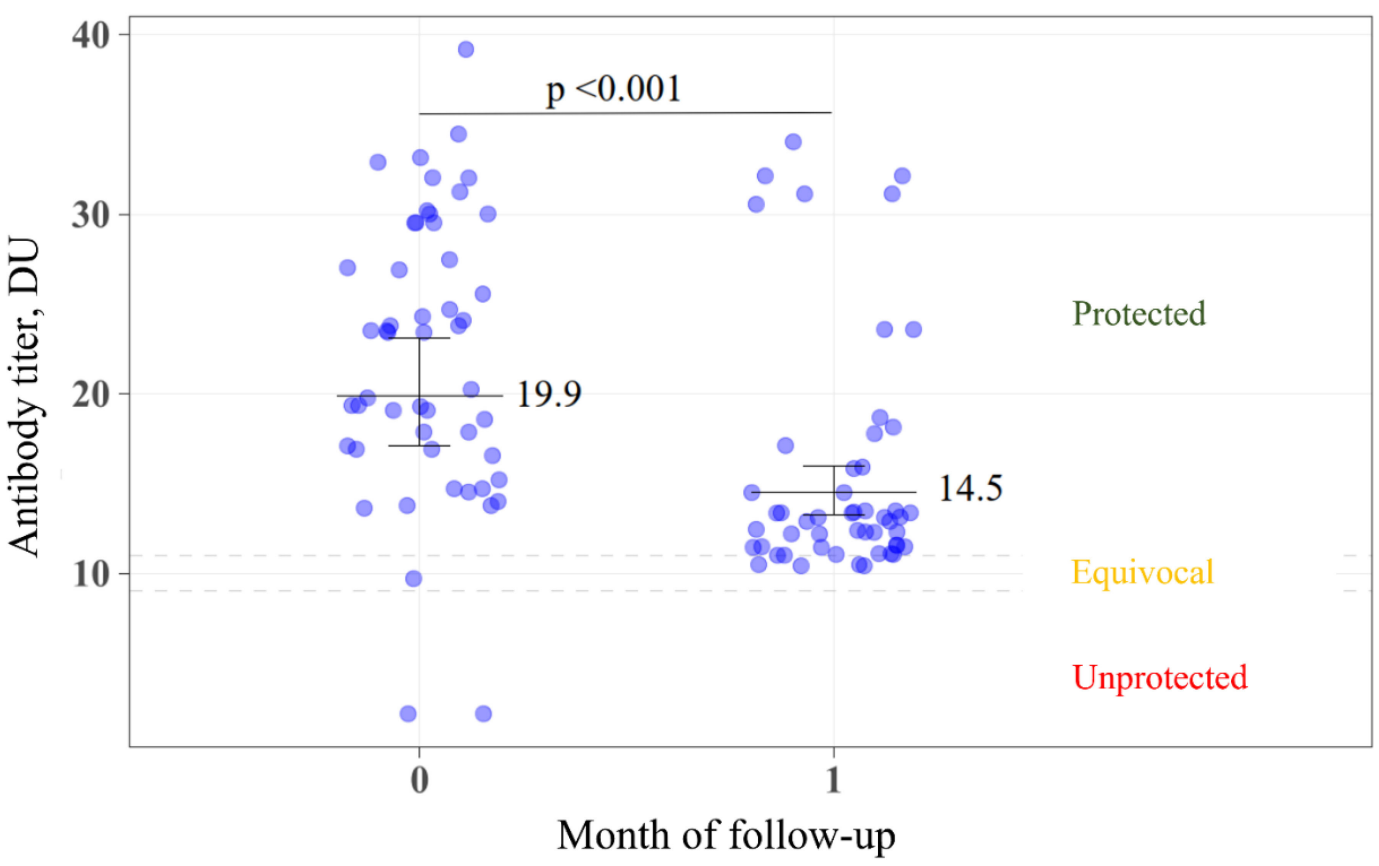
Geometric mean titers (GMT) of IgG antibodies against Bordetella pertussis in adolescents before and one month after revaccination with the Tdap vaccine. The central horizontal line represents the GMT, and the vertical bars indicate the 95% confidence interval (95% CI). Points on the plot represent individual antibody titers of participants.

**Figure 8:**
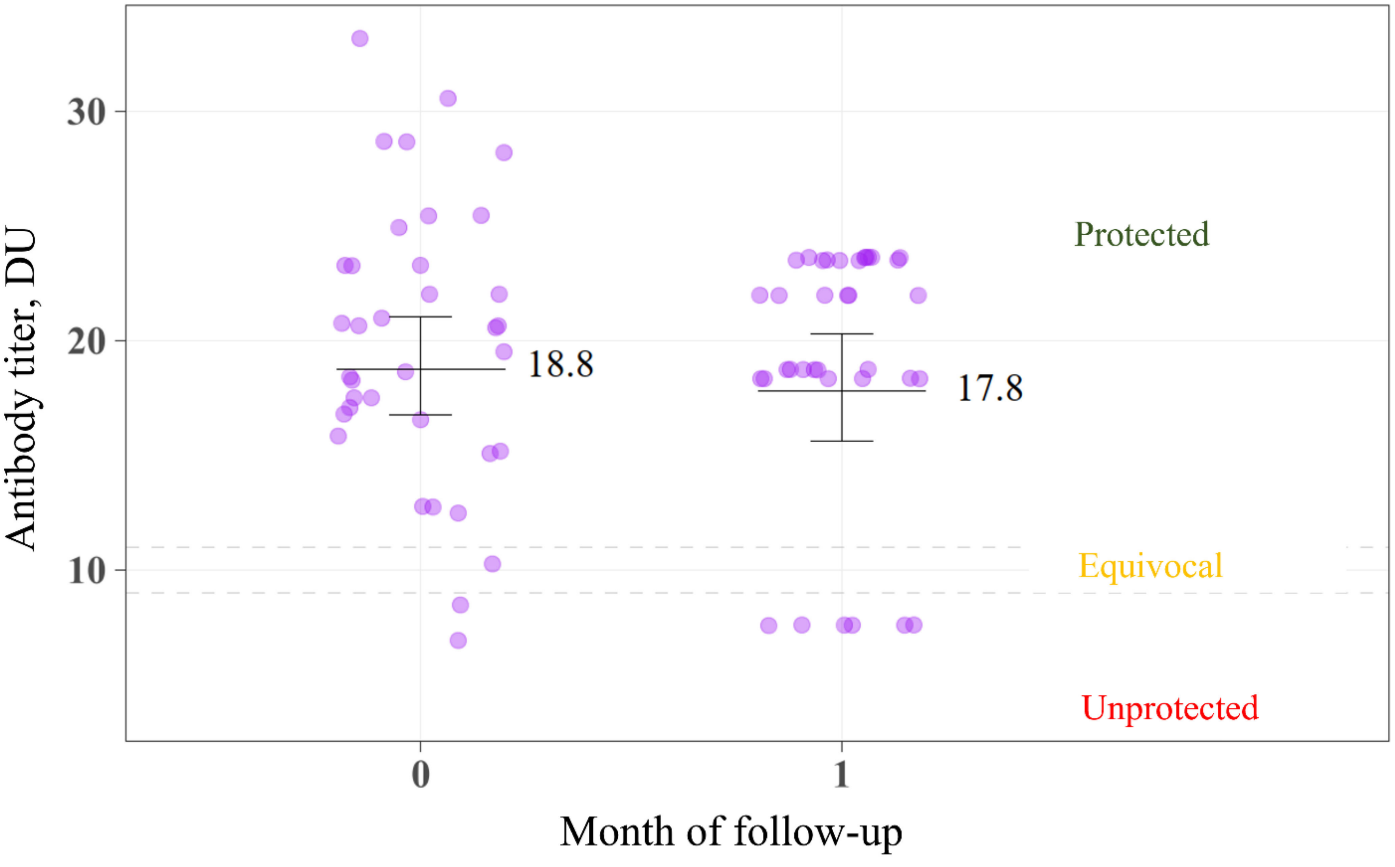
Geometric mean titers (GMT) of IgG antibodies against Bordetella pertussis in adolescents before and one month after revaccination with the Tdap-М vaccine. The central horizontal line represents the GMT, and the vertical bars indicate the 95% confidence interval (95% CI). Points on the plot represent individual antibody titers of participants.

Analysis of geometric mean titers (GMTs) of IgG antibodies against Bordetella pertussis showed that in adolescents revaccinated with Tdap, GMT decreased by the first month of follow-up from 19.9 IU/mL to 14.5 IU/mL (p < 0.001) (**Table 8**, **Figure 7**). In the Tdap-М group, baseline antibody levels against pertussis (18.8 IU/mL) slightly decreased to 17.8 IU/mL, although within-group comparison did not reveal a statistically significant change. Nevertheless, antibody levels in this group remained significantly higher than in adolescents who received Tdap (17.8 IU/mL vs 14.5 IU/mL; p < 0.016) (**Table 8**, **Figures 7** and **8**).

**Table 8:** Geometric mean titers (GMT) of IgG antibodies against Bordetella pertussis in adolescents before and one month after revaccination with different vaccines.

Vaccine	n	Month 0 (baseline)	Month 1 (post-vaccination)	p*
		GMT IU/mL (log2 ± SD)	GMT IU/mL (log2 ± SD)	
Tdap	51	19.9 (log2 4.31 ± 0.79)	14.5 (log2 3.86 ± 0.49)	< 0.001
Tdap-М	36	18.8 (log2 4.23 ± 0.50)	17.8 (log2 4.15 ± 0.58)	ns
P**		Ns	0.016	

Data are presented as geometric mean titers (GMT), IU/mL (log_2_(GMT) ± SD [log_2_(titer)]).

* Paired t-test.

** Independent t-test.

### 3.4 Assessment of vaccine tolerability

Local reactions in the form of injection-site induration were observed in 6 of 34 adolescents (17.6%) after administration of Td-М and in 10 of 51 adolescents (19.6%) after administration of Tdap No local reactions were observed in adolescents who received Tdap-М (n = 36). As a result, statistically significant differences were identified between the groups (p < 0.01).

Among systemic reactions during the post-vaccination period, mild fever (>37.0–≤37.5 °C) was recorded in 2 (5.9%), 5 (9.8%), and 5 (13.9%) cases following administration of Td-М, Tdap, and Tdap-М, respectively, without statistically significant differences between the groups. Fatigue was reported in one case (2.9%) after АДС-М and in two cases (5.6%) after administration of Tdap-М. Headache was also rare, occurring in isolated cases: 1 (2.9%) after Td-М, 2 cases (2.0%) after Tdap, and 2 cases (5.6%) after Tdap-М.

Both local and systemic reactions occurred during the first days of the post-vaccination period and were assessed as mild in severity.

## 4 Discussion

Worldwide, booster doses of vaccines are recommended for adolescents, adults, and pregnant women in order to enhance protection against diphtheria and tetanus infections. However, according to data from the World Health Organization on immunization (as of March 7, 2026), only 130 countries or regions have implemented diphtheria revaccination programs for adolescents aged 9 to 18 years, and 117 for pregnant women. Russia adheres to the principles of global medical practice and recommends a second revaccination against diphtheria and tetanus at school age (7 years), a third at adolescence (14 years), and further revaccination in adulthood at 10-year intervals.

With regard to pertussis infection, since the late 1950s, an adsorbed vaccine against pertussis, diphtheria, and tetanus (DTwP) has been used in Russia for population prevention. Primary immunization with the DTwP vaccine in Russia until 2021 consisted of three doses administered at the ages of 3, 4, 5, and 6 months. Within this immunization strategy, vaccination coverage among children aged 12 months has not fallen below 95% of all children eligible for vaccination in the Russian Federation since 2005 (21). In contrast to diphtheria and tetanus, the current National Immunization Schedule of the Russian Federation provides for only one pertussis booster vaccination (at 18–24 months of age) and does not include additional booster doses. Revaccination of schoolchildren, adolescents, and adults with whole-cell pertussis-containing vaccines was discontinued in Russia in 1980. In this context, the studied group of 14-year-old adolescents is important for determining target cohorts for pertussis revaccination when updating the National Immunization Schedule.

The focus on adolescents is also justified from another perspective related to the demographic potential of the nation, since the establishment of robust post-vaccination immunity in this group is one of the guarantees of reducing the risk and preventing the development of infectious diseases and complications in the younger generation. There is no doubt that adolescents are future parents, and vaccination may contribute to the passive transplacental transfer of specific antibodies against many vaccine-preventable viral and bacterial infections, such as measles, rubella, mumps, pertussis, and others (22–24). At the same time, revaccination against diphtheria, tetanus, and pertussis at the age of 14 years can be considered one of the last opportunities to achieve high immunization coverage within organized groups, where the largest number of children can be reached.

Analysis of baseline data in 121 adolescent boys aged 14 years, who had previously received a second revaccination against diphtheria and tetanus at 7 years (6th dose) and a first revaccination against pertussis at 18–24 months (4th dose), showed that all (100% of cases) retained protection against tetanus, while only 2 (1.7%) lacked protective IgG levels against diphtheria toxoid, and 6 (6.9%) of 87 examined children lacked protection against B. pertussis. This indicates that this cohort of adolescents meets the criteria of epidemiological well-being adopted in the Russian Federation, according to which the proportion of unprotected individuals in the examined group should not exceed 5% for diphtheria and tetanus and no more than 10% seronegative for pertussis.

Results obtained in other countries, for example in China, where revaccination against diphtheria and tetanus is not carried out in adolescents and adults, as well as during pregnancy, and children receive a total of five doses of diphtheria/tetanus vaccine, with the last dose at 6 years, showed in a seroepidemiological study that the overall seropositivity rates were 61.82% (95% CI: 58.14, 65.39) and 71.61% (95% CI: 68.3, 74.92), respectively (25). The authors concluded that antibody concentrations against diphtheria and tetanus decreased with age and were lowest and below the required protective level in individuals older than 14 years, indicating an urgent need to revise the existing vaccination schedule.

A study of serum antibodies against diphtheria and tetanus in adolescents in Laos showed that 25.8% had antibody levels corresponding to protection against diphtheria, and 30.9% had sufficient immunity against tetanus (26). The authors suggest that the low level of protection may be due to insufficient vaccination coverage or waning immunity, indicating the need for booster vaccination before adolescence.

Insufficient protection against diphtheria among adolescents and adults is also demonstrated by studies in Vietnam, where even one month after revaccination with diphtheria and tetanus toxoids, protective antibody levels were observed in 81.0%–98.5%, respectively, and after 5 years these levels persisted in 72.7%–96.7% of cases (27). These findings indicate the high effectiveness of primary vaccination (4 doses of DTwP vaccine from 3 months to 18–24 months) and the second revaccination with Td-M toxoid at 7 years in establishing robust antitoxic immunity.

Of concern is the high level of herd immunity against pertussis among adolescents, with 93.1% (81 of 87 individuals) having protective IgG levels to B. pertussis. Typically, post-vaccination immunity to pertussis significantly wanes or is lost by school age, and in the absence of additional booster doses, older siblings and adults-parents and grandparents become the main source of infection for infants (28). In this regard, the use of reduced-antigen-content acellular vaccines against diphtheria, tetanus, and pertussis, approved for revaccination in individuals aged 4 to 64 years, allows protection of schoolchildren and adults at risk and contributes to increased protection of infants. However, in the Russian Federation, the last (4th) dose of pertussis vaccine is administered at 18–24 months, and it cannot be excluded that many children may have experienced asymptomatic pertussis before the age of 14, despite the exclusion of adolescents with documented infection from the study.

The possible association between high IgG levels to B. pertussis and prior infection is supported by studies in Brazilian volunteers, which showed that younger populations (4–9, 10–14, and 15–19 years) had higher antibody levels compared to adults and elderly individuals (20–39, 40–59, and ≥ 60 years), suggesting that pertussis predominantly circulates among adolescents and young adults (29).

Recent data from a systematic review using multiple diagnostic approaches indicate a significant prevalence of asymptomatic pertussis infection among adults and the elderly, suggesting their role in disease transmission (30).

Therefore, monitoring of post-vaccination immunity and scientific justification for introducing an additional pertussis booster in the Russian Federation is relevant for determining vaccination strategies for this infection (31).

The third revaccination against diphtheria and tetanus administered to adolescents at 14 years of age using different vaccines resulted in the formation of IgG against tetanus at high (>1.0 IU/mL) levels in 100% of cases, ensuring long-term protection. The highest GMT was observed in the group receiving Td-М toxoid (8.5 IU/mL), which significantly exceeded the values in groups revaccinated with Tdap (5.4 IU/mL; p<0.001) and Tdap-М (6.1 IU/mL; p<0.001), although it should be noted that the tetanus toxoid content in Td-M and Tdap-M vaccines is identical ((5(20 Lf(IU))), and even higher ((10(≥40) Lf(IU))) in the Tdap vaccine. A possible role of aluminum adjuvants and their concentration per vaccine dose cannot be excluded, as the aluminum hydroxide content is 0.55 mg in the Td-M vaccine, compared with 0.30 mg and 0.33 mg in the comparator vaccines (32, 33).

Despite a significant increase in IgG to diphtheria toxoid one month after revaccination, without differences between the observed groups, the proportion of individuals with high (>1.0 IU/mL) antibody levels ensuring long-term protection was lower than for tetanus toxoid and amounted to 82.4%, 64.7%, and 61.1% after administration of Td-М toxoid, Tdap and Tdap-М vaccines, respectively. An advantage of using the vaccine containing only diphtheria and tetanus toxoids was also identified through analysis of Pearson standardized residuals, which showed that adolescents receiving Td-М Td toxoid more frequently developed high levels of long-term protective antibodies to diphtheria toxoid (std.res. = 2.03). At the same time, the number of children with only sufficient levels of protection (std.res. = −2.03) was two times lower than in groups revaccinated with Tdap and Tdap-М.

A different pattern was observed for post-vaccination IgG to B. pertussis in adolescents one month after revaccination, where an increase to protective levels (>11 DU) was noted only in seronegative (<9 DU) individuals and those with equivocal antibody levels (9–11 DU), regardless of the vaccine used. In the post-vaccination period, no significant differences were observed within or between groups in the proportion of individuals protected (>11 DU) against pertussis, which amounted to 88.2% versus 94.1% baseline after Tdap and 83.3% versus 91.7% after Tdap-М.

It should be noted that in adolescents revaccinated with the Tdap vaccine, which contains higher antigen quantities (25 μg pertussis toxin and 25 μg filamentous hemagglutinin), a decrease in GMT IgG to B. pertussis was observed after one month (from 19.9 IU/mL to 14.5 IU/mL; p<0.001). In contrast, in those vaccinated with reduced-antigen Tdap-М (containing 2.5 μg pertussis toxin, 5 μg filamentous hemagglutinin, 5 μg fimbriae types 2 and 3, and 5 μg pertactin), antibody levels remained stable (17.8 IU/mL vs 18.8 IU/mL baseline) and were higher than in the Tdap group (p<0.016). The advantage of Tdap-М is also reflected in systematic reviews demonstrating that, despite lower antigen content, these vaccines are more effective than monocomponent B. pertussis vaccines and have a favorable safety profile (34, 35). Thus, our data show that even in the presence of initially high levels of specific IgG antibodies to B.pertussis, the use of the vaccine with a reduced antigen content is advantageous over a vaccine with a high antigen content per dose. However, the latter has the advantage of being used in infants to establish primer immunity.

Similar results have been reported after a single dose of Tdap-М in adolescents and adults, showing protective IgG levels against diphtheria (99%) and tetanus (100%), with seropositivity to B. pertussis antigens persisting for up to 10 years in most participants (36).

Other studies also report that one month after Tdap-М revaccination, nearly all adolescents and adults had IgG levels to diphtheria toxoid above 0.1 IU/mL, with only a slight decline to 95% after 5 and 10 years (37). High levels of IgG to tetanus toxoid persisted throughout the observation period. GMT IgG to B. pertussis antigens decreased after vaccination but remained above baseline at all time points, except for antibodies to pertussis toxin at 5 and 10 years, which declined to near pre-vaccination levels. Similar patterns were observed in adolescents and adults (38).

Thus, the obtained data on pertussis immunity indicate that 14-year-old adolescents do not require a second booster against this infection, as a high proportion (93.1%) already have protective immunity, who, with a high degree of probability, already have post-infection immunity at this age, and revaccination does not lead to a further increase in specific antibodies. This indicates that, strategically, it is necessary to carry out revaccination of preschool children, and then 14–15 year old. The presence of a high proportion of adolescents with high levels of antibodies to pertussis is evidenced by the data of other researchers from the Republic of Belarus, where the proportion of seropositive people to whooping cough among schoolchildren aged 7–14 years living in Minsk varied from 81.5% to 90.9% (39). According to Mayansky N.A. et al. (40), when studying pertussis immunity in schoolchildren aged 11–17 years in 7 regions of the Russian Federation, it was shown that 71.1% of children aged 15–17 years had protective immunity due to the disease they had suffered. According to Basov A.A (41). and others, in a study of pertussis immunity in schoolchildren aged 6–17 years living in the Moscow region, showed that the proportion of children seronegative to pertussis was 87.9% in the 15–17 age groups. It should be noted that in these countries, the second booster dose against whooping cough at 6–7 years of age is not administered as part of the National Immunization Schedule. It is likely more appropriate to administer a second pertussis booster at 6–7 years in combination with diphtheria and tetanus toxoids (42, 43).

Evaluation of the safety profile of the vaccines used showed that none were associated with unusual adverse events in the post-vaccination period. Expected systemic and local reactions were mild and included fever not exceeding 37.5 °C in 5.9%–13.9% of cases, fatigue and headache in isolated cases, and local induration in 17.6% and 19.6% of cases only among those revaccinated with Td-М toxoid and Tdap vaccine, indicating an advantage of Tdap use in adolescence.

A limitation of the present study is the absence of additional investigations to explain why no increase in IgG to B. pertussis was observed one month after revaccination in adolescents who initially had protective antibody levels. We suggest that the high levels of IgG to B. pertussis in adolescents reflect the formation of mixed “surrogate” immunity: primary immunization with DTwP promotes a Th1/17 response, while natural infection induces a long-lasting Th1/17 response and strong mucosal T-cell immunity, including sIgA. This may influence protection against subsequent infections and may also be associated with a transient refractory phase characterized by suppressed antibody synthesis following administration of acellular pertussis vaccines.

## 5 Conclusion

The analysis of the obtained data in 14-year-old adolescents regarding protection against diphtheria and tetanus after the second revaccination administered at 7 years of age demonstrates that this cohort does not represent a risk group for the development of unfavorable epidemiological situations for these infections. It was established that, in the absence of pertussis-containing vaccines during preschool and school age, adolescents exhibit a high level of protection (93.1%) against B. pertussis, which indicates natural boosting due to the circulation of the infection.

The third revaccination is associated with a significant increase in antitoxic antibodies against diphtheria and tetanus, regardless of the vaccine used. The second revaccination against pertussis administered to adolescents at 14 years of age is accompanied by the formation of IgG to B. pertussis only in seronegative individuals and those with equivocal antibody levels, a group that is small in number and accounts for 6.9%. Revaccination of 14-year-old adolescents who already have protective levels of antibodies against pertussis did not lead to a further increase in specific antibodies. At the same time, the administration of the Tdap vaccine with a reduced antigen content, in contrast to the Tdap vaccine, has an advantage, as it does not lead to a decrease in GMT IgG to B. pertussis during one month of follow-up. The revaccination doses of the vaccines used demonstrated a favorable safety profile.

Thus, the feasibility of administering a third revaccination against diphtheria and tetanus in adolescents is supported, while the necessity of a second revaccination against pertussis remains questionable.

## Data Availability

The original contributions presented in the study are included in the article/supplementary material. Further inquiries can be directed to the corresponding author.
